# Formulation of Intranasal Mucoadhesive Thermotriggered In Situ Gel Containing Mirtazapine as an Antidepressant Drug

**DOI:** 10.3390/gels9060457

**Published:** 2023-06-02

**Authors:** Mohammed Ghazwani, Rajalakshimi Vasudevan, Geetha Kandasamy, Naredla. Manusri, Praveen Devanandan, Ranadheer Chowdary Puvvada, Vinoth Prabhu Veeramani, Premalatha Paulsamy, Krishnaraju Venkatesan, Kumarappan Chidmabaram, Rajeshri Dhurke

**Affiliations:** 1 Department of Pharmaceutics, College of Pharmacy, King Khalid University, Abha 62529, Saudi Arabia; 2 Department of Pharmacology and Toxicology, College of Pharmacy, King Khalid University, Abha 62529, Saudi Arabia; 3 Department of Clinical Pharmacy, College of Pharmacy, King Khalid University, Abha 62529, Saudi Arabia; 4 Department of Pharmaceutics, St. Peter’s Institute of Pharmaceutical Sciences, Hanamkonda 506001, Telangana, India; 5 Department of Pharmacy Practice, St. Peter’s Institute of Pharmaceutical Sciences, Hanamkonda 506001, Telangana, India; 6 Department of Pharmacy Practice, Faculty of Pharmacy, University of Tabuk, Tabuk 71491, Saudi Arabia; 7 College of Nursing, King Khalid University, Abha 62529, Saudi Arabia

**Keywords:** intranasal, thermosensitive gels, mirtazapine, nose to brain delivery, nanoemulsions, antidepressant

## Abstract

The purpose of the present work was to develop nanoemulsion-based formulations of mirtazapine for intranasal delivery using a spray actuator to target the brain for treating depression. Research on the solubility of medications in different oils, surfactants, co-surfactants, and solvents has been done. Using pseudo-ternary phase diagrams, the various ratios of the surfactant and co-surfactant mix were computed. Thermotriggered nanoemulsion was formulated using different concentrations of poloxamer 407 (i.e., 15%, 15.5%, 16%, 16.5% up to 22%). Similarly, mucoadhesive nanoemulsion using 0.1% Carbopol and water-based plain nanoemulsions were also prepared for comparative assessment. The developed nanoemulsions were analyzed for physicochemical properties, i.e., physical appearance, pH, viscosity, and drug content. Drug-excipient incompatibility was determined by Fourier transform infrared spectral (FTIR) analysis and differential scanning calorimetry (DSC). In vitro drug diffusion studies were conducted for optimized formulations. Among the three formulations, RD1 showed the highest percentage of drug release. Ex vivo drug diffusion studies were conducted on freshly excised sheep nasal mucosa with Franz diffusion cell simulated nasal fluid (SNF) for all three formulations up to 6 h, and the thermotriggered nanoemulsion (RD1) showed 71.42% drug release with 42.64 nm particle size and a poly dispersity index of 0.354. The zeta potential was found to be −6.58. Based on the above data, it was concluded that thermotriggered nanoemulsion (RD1) has great potential to be used as an intranasal gel for treating depression in patients. It can offer great benefits by reducing dosing frequency and improving bioavailability of mirtazapine by direct nose-to-brain delivery.

## 1. Introduction

Intranasal delivery as a non-invasive drug delivery method has gained popularity in recent years. A wide range of medicinal chemicals may be delivered intranasally for topical, systemic, and central nervous system action since the nasal mucosa provides many advantages as a target tissue for drug delivery. It is currently acknowledged that intranasal drug delivery is a beneficial and trustworthy option compared to oral and parenteral routes. Without a doubt, intranasal medication administration has been utilized extensively for a long time for the symptomatic alleviation, prevention, and treatment of topical nasal diseases [1,2] The nasal mucosa has, however, recently made a significant comeback as a therapeutically effective route for systemic drug administration. In general, pharmacologically active substances with limited stability in gastrointestinal fluids, poor intestinal absorption, and/or significant hepatic first-pass elimination, such as peptides, proteins, and polar medicines, are among the main targets for intranasal delivery. The intranasal delivery method appears to be an effective strategy to get over the blood-brain barrier (BBB) barriers, enabling direct medication delivery of central nervous system (CNS)-active substances during their biophase [3,4].

A nanostructured drug delivery system known as a nanoemulsion (NE) is characterized by its small droplet size and kinetic stability, which prevents flocculation even when the substance is stored for extended periods of time. It is suitable for drug delivery because it has a number of benefits, such as ease of synthesis and optimization, high drug loading capacity, higher drug permeability across mucosal membranes, and increased bioavailability of drug molecules [5,6,7,8].

Antidepressant mirtazapine is used to treat moderate to severe depression. The Food and Drug Administration has approved it as the sole tetracyclic antidepressant to treat depression and anxiety. Being a powerful antagonist at postsynaptic 5-HT2 and 5-HT3 (serotonergic) and central noradrenergic receptors, it is also used to treat anxiety by boosting central noradrenergic and serotonergic (5-HT1) neurotransmission [9,10]. Although mirtazapine is quickly absorbed after oral administration, its absolute bioavailability is only 50% due to strong first-pass metabolism. These factors highlight the necessity for an alternate drug delivery technology that can target mirtazapine to the brain with precision. Intranasal administration methods may be predicted to decrease the extensive dispersion of medications to non-targeted areas such systemic/peripheral circulation since pharmaceuticals are preferentially transported to the brain. To ensure that the drug is quickly transported through the nasal mucosa and via the olfactory bulb, the delivery mechanism must be carefully constructed [11,12]. The preparation and characterization of a thermotriggered nanoemulsion loaded with mirtazapine for intranasal administration was the goal of this work. The research was done with the intention of delivering drugs to the brain for a faster onset of action than oral administration, minimizing adverse effects, maximizing therapeutic index, and lowering dosage and dosing frequency.

## 2. Result and Discussion

### 2.1. Saturation Solubility Studies

**Figure 1:**
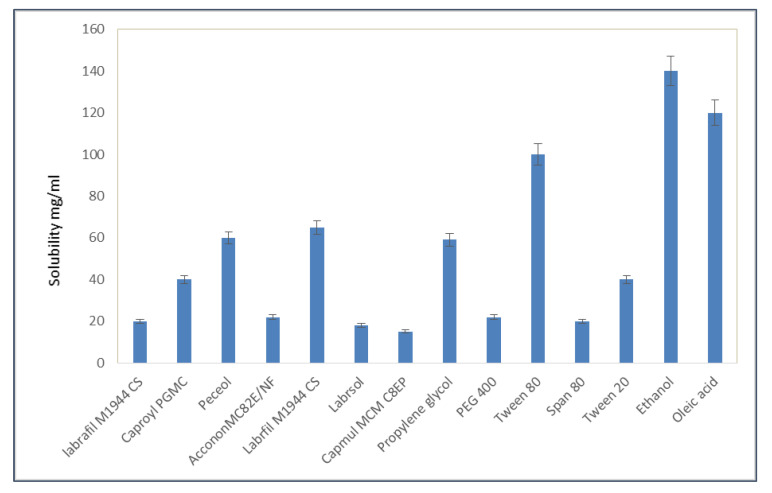
Saturation solubility studies of mirtazapine in different solvents and oils.

The saturation solubility studies of mirtazapine were carried out in different solvents. Oleic acid was selected as the oil phase from the solubility studies (Figure 1) based on the drug’s solubility, and Tween 80 and ethanol were selected as the surfactant and co-surfactant, respectively. Tween 80 not only effectively dissolves drugs but also effectively forms emulsions.

### 2.2. Construction of Pseudo-Ternary Phase Diagrams

**Figure 2:**
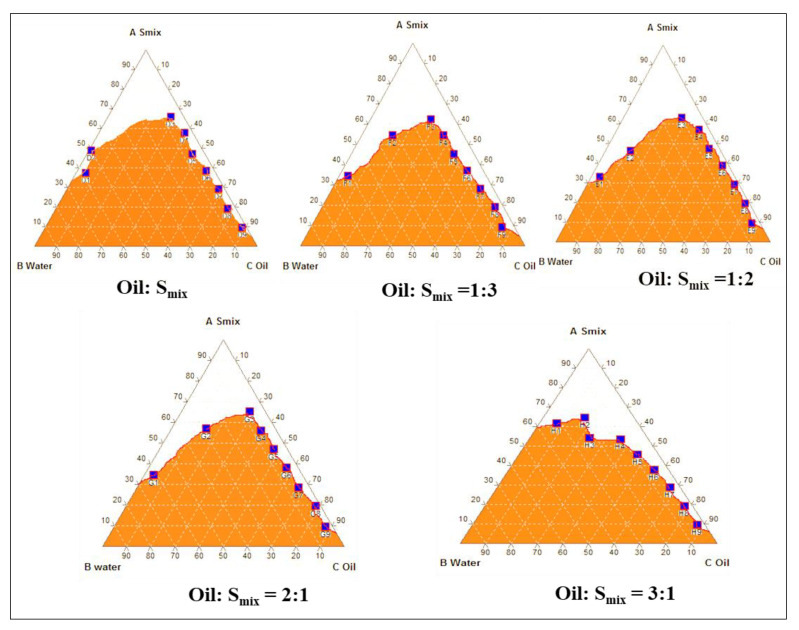
Pseudoternary phase diagrams.

Microemulsions were prepared using oleic acid as the oil phase, Tween 80 as the surfactant, and ethanol as the co-surfactant. Figure 2 represents the pseudo-ternary phase diagrams of oleic acid with various ratios of Tween 80 and ethanol. Based on the pseudo-ternary phase diagrams, a higher ratio of Smix than oil, i.e., 9:1 ratio of Smix:oil was selected. The optimized ratio mixture of surfactant to co-surfactant was 1:3.

### 2.3. Development of Microemulsion Formulations

Formulations were developed based on the highest microemulsion zone obtained from pseudo-ternary phase diagrams. A 1:3 ratio of Smix (Tween 80: ethanol) and a 9:1 ratio of oil and Smix was selected, and further various formulations like RD1, RD2, RD3 were formulated. Carbopol 934P was used as a mucoadhesive agent in the formulation, which helps the formulation to remain adhered to the mucosal membrane. A thermotriggered formulation was prepared by changing different concentrations of poloxamer 407, i.e.,15%, 16%, 16.5%, 17%, 17.5%, 18%, 18.5%, 19%, 19.5%, and 20%. Poloxamer 407 of 18.5% concentration was selected based on gelling time and gelling temperature. The optimized formulation (RD1) gave a gelling time of 4 sec and a gelling temperature of 32 °C.

### 2.4. Characterization of Nanoemulsion

#### 2.4.1. Physicochemical Properties

All the developed nanoemulsion formulations were physically examined and were confirmed to be clear and transparent. The pH of all the formulations was measured using a pH meter (Remi Equipment Pvt. Ltd., Kolkata, India) and was found to be close to nasal pH, indicating an acceptable range, as evident from Table 1.

Thermotriggered nanoemulsion gel formation: The thermotriggered nanoemulsion formulation was added dropwise into distilled water by heating. A consistent thermal gel was formed at 32 °C and was stable.

#### 2.4.2. In Vitro Drug Diffusion Studies

**Figure 3:**
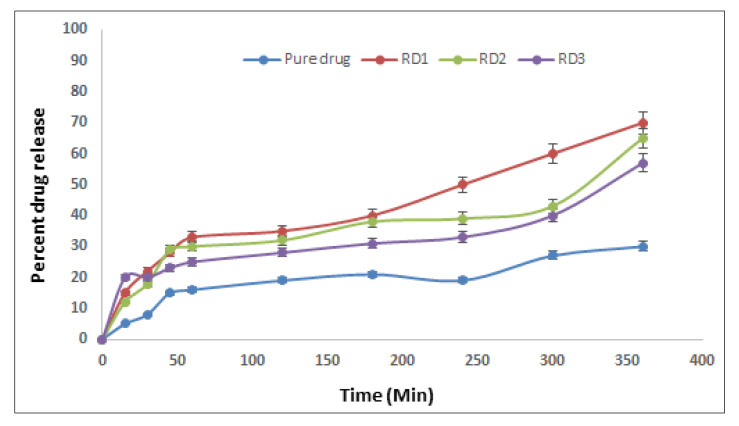
In vitro diffusion profiles of the formulations.

For the created formulations, in vitro release tests were carried out utilizing Franz diffusion cells and diffusion experiments. The study was carried out for up to 6 h for developed thermotriggered nanoemulsion (RD1), mucoadhesive nanoemulsion (RD2), and water-based nanoemulsion (RD3). The results are graphically represented in Figure 3. It can be observed that optimal formulation, i.e., thermotriggered nanoemulsion (RD1), has shown 71.42% drug release after 6 h, whereas mucoadhesive nanoemulsion (RD2) and water-based nanoemulsion (RD3) showed drug releases of 66.12% and 60%, respectively, after 6 h. This indicated that the thermotriggered nanoemulsion (RD1) was better among the three formulations. The high release may be because of the poloxamer 407, which is helping the formulation to form in situ gel as soon as it is sprayed in the nose, while the mucoadhesive properties of carbopol are keeping the nanoemulsion adhered to the mucosal lining. Formulation RD2 lacks poloxamer 407, hence it doesn’t get sufficient time for contact with the mucosal area and drains. The least release with RD3 formulation is due to the absence of a mucoadhesive polymer as well as a thermosensitive polymer.

#### 2.4.3. Ex Vivo Permeation Studies

**Figure 4:**
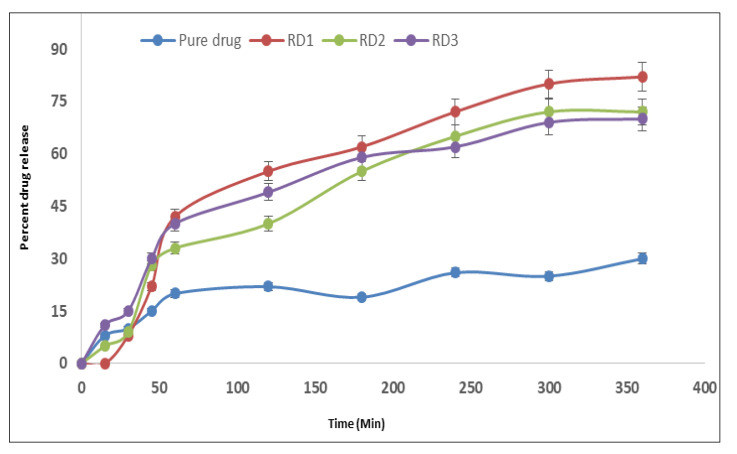
Ex vivo diffusion profiles of the formulations.

Figure 4 illustrates how mirtazapine thermotriggered nanoemulsions (RD1), mucoadhesive nanoemulsions (RD2), and water-based nanoemulsions (RD3) permeate the nasal mucosa. From the figure, it is evident that pure drug dispersion could show only 25% drug difussion in 6 h, whereas the nanoemulsion formulation showed greatly improved drug diffusion compared with the pure drug. Formulation RD1 showed drug diffusion of more than 80%, whereas RD2 and RD3 only showed drug diffusion in the range of 70–72%. Due to a lack of mucoadhesion and thermosensitive characteristics, the drug in the water-based nanoemulsion (RD3) demonstrated the lowest permeability. As the contact time with the mucosal area was extended, drug penetration in mucoadhesive and thermotriggered nanoemulsions improved. The high transcellular uptake, high solubilization capacity, possibility for improved absorption by gel formation by mucoadhesion, and presence of a poloxamer may all contribute to the higher permeability. Carbopol 934 P may have also improved drug absorption; this may be because it opened tight junctions, which made it easier for pharmaceuticals to go through paracellular pathways [13].

#### 2.4.4. Fourier Transform Infrared Spectroscopy (FTIR)

**Figure 5:**
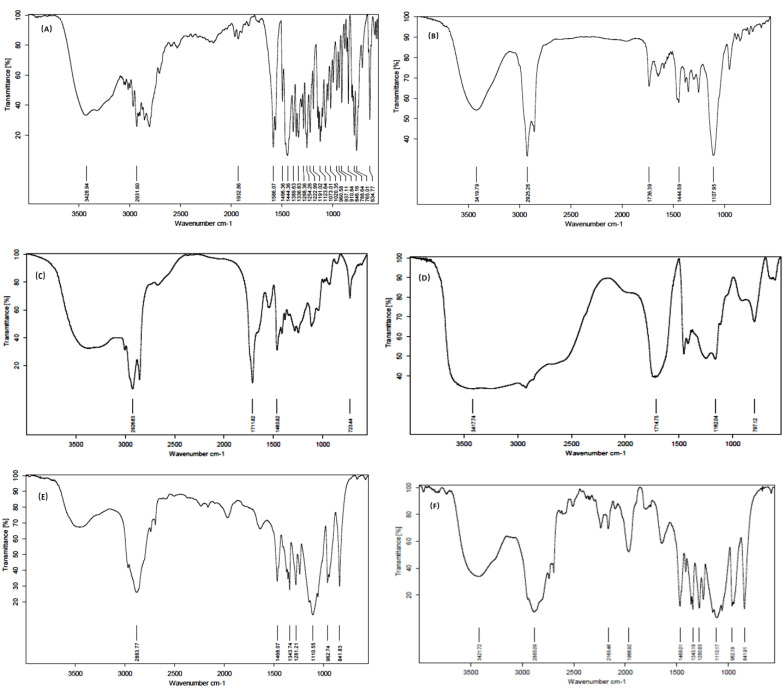
IR Spectra of (**A**) mirtazapine, (**B**) formulation, (**C**) oleic acid, (**D**) Carbapol934P, (**E**) poloxamer 407, and (**F**) PEG 6000.

FTIR studies were performed to determine the compatibility between pure drug and excipients. FTIR spectrum of pure mirtazapine, thermotriggered formulation, and poloxamer 407 characteristic peaks are given in Figure 5. From the results, it was concluded that there is no considerable change in the drug when mixed with the excipients. It can be concluded that there is no drug excipient incompatibility.

#### 2.4.5. Differential Scanning Calorimetry (DSC)

DSC studies were performed for pure mirtazapine, thermotriggered nanoemulsion, poloxamer 407, and other excipients used in the formulation to know the thermal behavior and the physical state of the drug in the formulation (crystalline/amorphous) through characteristic melting points.

**Figure 6:**
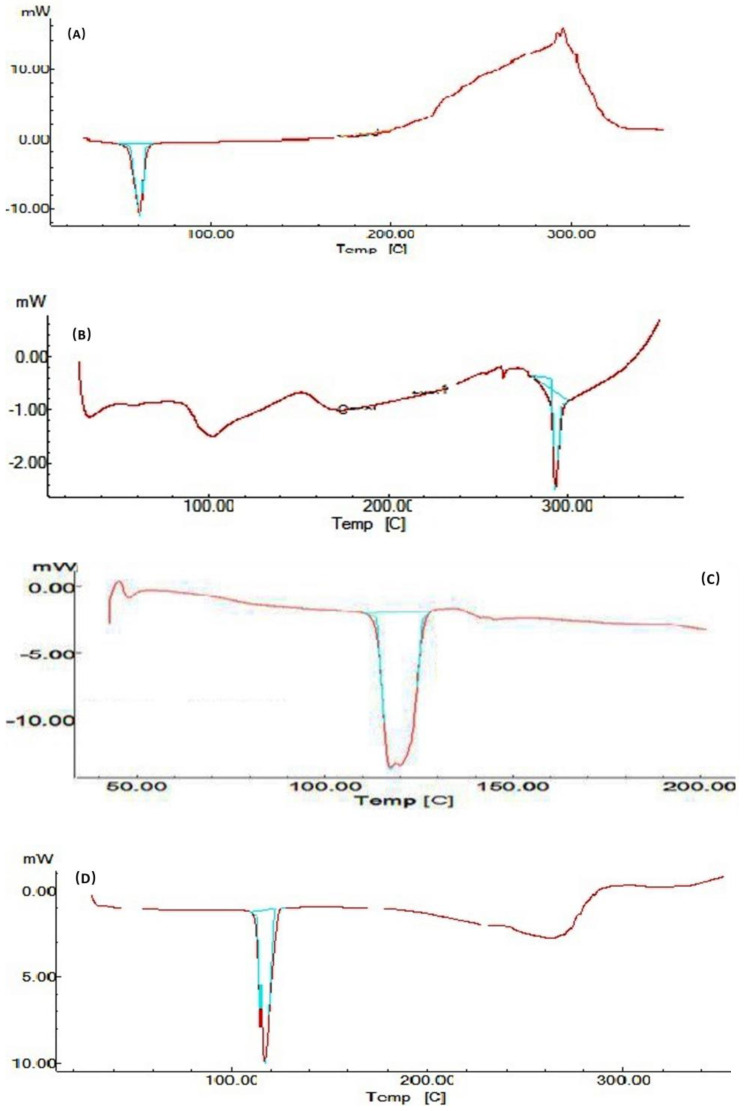
Reports of DSC thermograms of pure drug, formulation, and excipients. (**A**) Poloxamer 407, (**B**) Oleic acid, (**C**) Formulation, (**D**) Mirtazapine.

The DSC thermogram of mirtazapine, thermotriggered nanoemulsion, and other excipients are shown in Figure 6. The thermogram of pure mirtazapine showed melting endotherm at 116 °C. The thermogram of thermotriggered nanoemulsion showed an endotherm peak at 162.85 °C, and poloxamer 407 showed an endotherm peak at 60.53 °C, which indicates that there is no significant change in the melting endotherm of the pure drug. The slightly broad peak in the formulation indicates the change in the state of the drug to an amorphous state.

#### 2.4.6. Measurement of Particle Size and Zeta Potential

**Figure 7:**
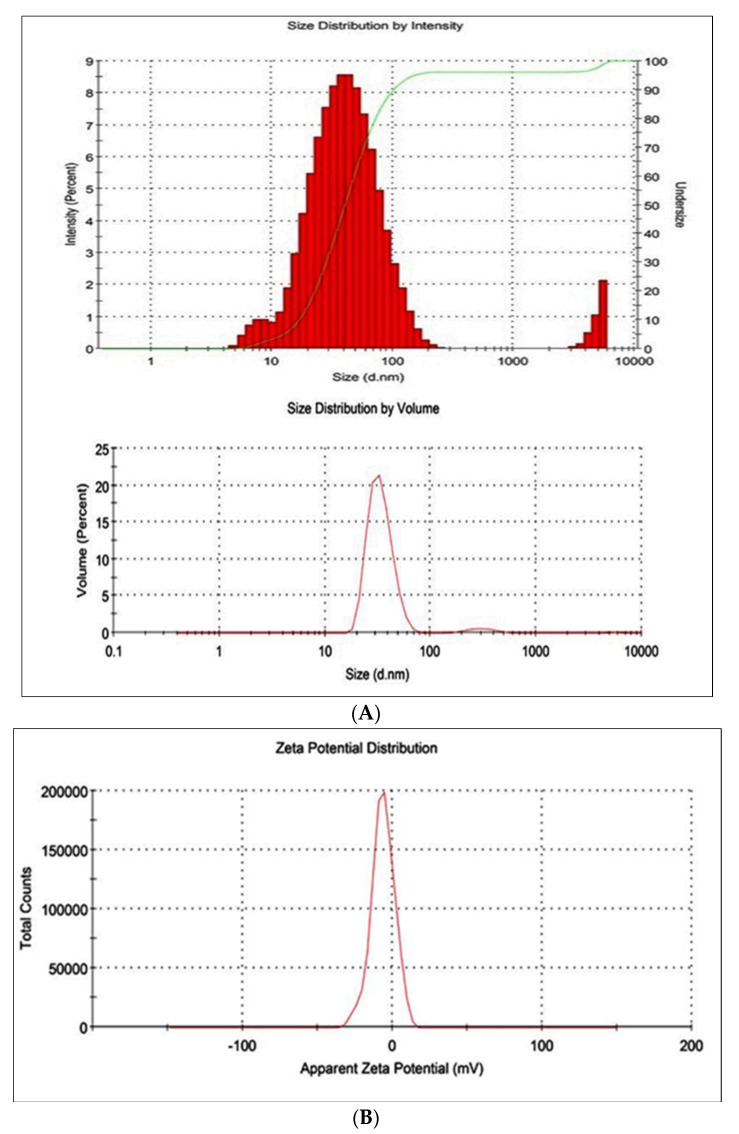
Particle size data of thermotriggered nanoemulsion (RD1), (**A**) size distribution and (**B**) zeta potential.

The average sizes of the globules of RD1, RD2, and RD3 were found to be 42.6 nm, 135.2 nm, and 52.44 nm, respectively, as seen in Table 2 and depictated in Figure 7. The percentage of the intensity of distribution of the 42.64 nm-sized globules of thermotriggered nanoemulsion was high, i.e., 90.2%, whereas the average globule size and distribution percentage of RD2 and RD3 were found to be 50.8 and 60.5, respectively.

#### 2.4.7. Transmission Electron Microscopy (TEM)

**Figure 8:**
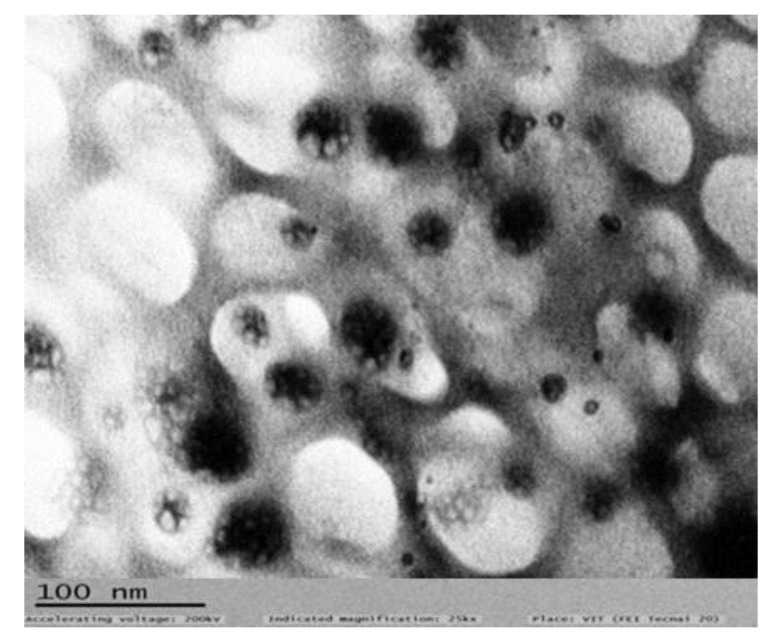
TEM Photographs of Thermotriggered Nanoemulsion (RD1).

From the images obtained by TEM analysis, it is evident that thermotriggered nanoemulsion (RD1) showed highly uniform and spherical shapes globules. White, nearly spherical globules were seen against a dark background created by negative staining with phosphotungstic acid, as evident from Figure 8.

## 3. Stability Studies

Nanoemulsions are considered to be thermodynamically stable systems that are formed with a particular concentration of oil, surfactant, and water, with no phase separation, creaming, or cracking. Selected formulations from the phase diagram were subjected to different stress stability tests, such as a heating-cooling cycle, centrifugation, and a freeze-thaw cycle. All nanoemulsion formulation cycles were found to be stable.

## 4. Statistical Analysis

The results of different formulations were analyzed by one-way ANOVA. The values were considered to be statistically significant when the *p* value was less than 0.05. It was observed that the *p* value of all the responses was found to be below 0.05, hence considered as significant.

## 5. Conclusions

Thermotriggered intranasal gel for the antidepressant mirtazapine was successfully developed to treat depression in patients. Three different nanoemulsions, including thermotriggered nanoemulsion (RD1), mucoadhesive nanoemulsion (RD2), and plain nanoemulsion, were developed, and comparative studies were done. After comparison of these three formulations for various physicochemical and analytical parameters, it was concluded that the thermotriggered nanoemulsion (RD1) consisting of carbopol and PEG 6000 was best suitable for intranasal delivery in terms of properties like excellent globule size, shape, zeta potential, polydispersity index, and percentage of drug release. From the results obtained in ex vivo studies conducted on freshly excised sheep nasal mucosa, it can be hypothesized that the thermotriggered nanoemulsion may have still better absorption capacity through the olfactory and trigeminal nerves, as it shows good paracellular and transcellular transport and can be efficiently used to treat major depressive disorder. To support the hypothesis, in vivo animal studies must be done; these studies would give a clear idea on brain-targeting efficiency of the formulation.

## 6. Materials

Mirtazapine (CAS: 85650-52-8) was procured as a kindly gifted sample from Wockhardt Pvt. Ltd., Aurangabad, Maharashtra, India. Poloxamer 407 (CAS: 691397-13-4) was obtained as a gifted sample from BASF India, Ltd., Mumbai, India. Labrafil M1944 CS (CAS: 69071-70-1), Capryol PGMC (CAS: 31565-12-5), Peceol, and Labrosol, were gift samples from Gattefosse India, Pvt. Ltd. (Mumbai, India), while Campul MCM C8 EP (CAS: 26402-26-6) and Acconon MC82E/NF (CAS: 85536-01-8) were a generous gifts from Abitech Ltd. Finar Chemicals, Hyderabad, India supplied the Tween 80, Tween 20, Span 80, propylene glycol, polyethylene glycol 400, and oleic acid. The remaining reagents were all of analytical grade.

## 7. Methods

### 7.1. Saturation Solubility Studies

The solubility studies of mirtazapine were carried out in different solvents and buffers, like double-distilled water, ethanol, dichloromethane, Labrafil M1944 CS, Caproyl PGMC, Peceol, Acconon MC82E/NF, Labrosol, Campul MCMC8EP, propylene glycol, PEG 400, Tween 80, Tween 20, Span 80, and oleic acid. In order to create saturated solutions of mirtazapine, excess amounts of the drug were added to 5 mL of each chosen vehicle and stirred on a rotary shaker for 48 h at 25 °C. After equilibrium was reached, samples were taken and centrifuged for 15 min at 10,000 rpm. Following the collection and proper dilution of an additional 100 L of supernatant with dichloromethane, mirtazapine samples were analyzed using UV-visible spectrophotometry at 228 nm [14].

### 7.2. Construction of Pseudo-Ternary Phase Diagram

Oleic acid was chosen as the oil phase, Tween 80 as the surfactant, and ethanol as the co-surfactant based on the solubility experiments. Tween 80 and ethanol were measured in the weight ratios (1:1, 1:2, 1:3, 2:1, and 3:1) of surfactant to co-surfactant (Smix). The pseudo-ternary phase diagrams were produced at room temperature by the water titration method. For each pseudo-ternary phase diagram, oil and Smix mixtures were created with weight ratios (*w*/*w*) of 1:9, 2:8, 3:7, 4:6, 5:5, 6:4, 7:3, 8:2, and 9:1. When the combination reached a particular point and began to break down into a macroemulsion, double-distilled water was gradually added to each ratio of Smix to oil while the mixture was being stirred magnetically. In order to complete the pseudo-ternary phase diagrams, the component concentrations were noted. The oil, surfactant, co-surfactant, and water contents were then chosen at the proper weight ratios based on the stability and transparency of the resulting microemulsions [14,15].

### 7.3. Development of Nanoemulsion Formulation

The criteria for selection of the microemulsion zone were carried out by observing the stability of the formulation from the pseudo-ternary phase diagrams. The microemulsion formulations were composed of S_mix_ (1:3) and oil. In accordance with the pseudo-ternary phase diagrams, a S_mix_ ratio of 1:9 was used. Oleic acid was used as oil in the microemulsion [14,15].

#### 7.3.1. Formulation of Thermotriggered Nanogel

Thermotriggered nanogel was prepared by a cold method, different concentrations of polymers were used, such as poloxamer 407 (10.5–20.5% *w*/*v*) Carbopol 934 P (0.1–0.5% *w*/*w*), and PEG 6000 (0.1–0.3% *w*/*v*) to determine the optimum concentration of poloxamer 407 and other polymers required for thermotriggered gelling. After a series of combinations, it was found that poloxamer 407 (18.5%), Carbopol (0.1%), and PEG 6000 (0.3%) were found to have optimum concentration, which formed thermotriggered gel at a temperature of 32 °C.

Thermotriggered nanogel was prepared by dissolving 0.1% Carbopol 934 P and 0.3% PEG 6000 in the aqueous phase by continuously stirring on a magnetic stirrer at 400 rpm. To the aqueous phase, 18.5% of poloxamer 407 was slowly added under continuous stirring in cold conditions [16]. This aqueous phase was then added dropwise to the nanoemulsions. After complete solubilization of poloxamer 407, the solution was stored at 4 °C overnight to get the thermotriggered nanogel.

Carbopol 934P was used as the mucoadhesive agent for its mucoadhesive properties to make the formulation adhere to the mucosal system; poloxamer 407 was used as the thermosensitive agent to form in situ gel after spraying into the nostrils. The active drug was added in the formulations depending upon the amount of formulation being delivered by the spray pump. Table 3 shows the composition of the nanoemulsions.

#### 7.3.2. Determination of Reproducibility of Dosage

The developed formulations were loaded into the 5 mL clean sterile glass container and were fitted with a spray actuator and crimpled.

The spray actuator was analyzed for determination of the reproducibility of the dosage. The formulation was sprayed in a test tube and weighed. The empty weight of the test tube was subtracted from the total weight. This was repeated a number of times, and the average weight of the dose was taken [17].

### 7.4. Characterization of Nanoemulsion

#### 7.4.1. Measurement of Droplet Size and Zeta Potential

Using the zetasizer, a dynamic light scattering or photon correlation spectroscopy technique was used to measure the mean droplet size and zeta potential (Malvern Instruments version 7.01). Using filtered, twice-distilled water, each was diluted to the proper concentration. Analysis of globule size was carried out at 25 °C and with a detection angle of 90 °C. Nanoemulsions’ size and polydispersity index were directly measured by the equipment [16,17,18].

#### 7.4.2. pH Measurement

A pH meter was used to determine the pH values of the nanoemulsion samples (Remi Equipment Pvt. Ltd., Kolkata, India). Before each usage, the pH meter was calibrated using buffer solutions having pH values of 4.0, 7.0, and 9.0. The formulation’s pH was measured three times, and the means of the measurements were computed.

#### 7.4.3. Measurement of Viscosity

A Brookfield viscometer with spindle no. DV-II+ PRO was used to measure the viscosities of nanoemulsions. The spindle code for LV1 is 61. The spindle was dipped in the preparation and revolved for 5 min at 100 rpm at room temperature.

### 7.5. Determination of Entrapment Efficiency

#### 7.5.1. Drug Content

Drug content was estimated by placing one dose (7.5 mg) equivalent microemulsion formulation in a 100 mL volumetric flask and adding a small volume of methanol, which solubilizes the microemulsion. Finally, the volume was made up to 100 mL, with a pH 6.4 phosphate buffer. It was shaken well for about 1 h in a shaker followed by centrifugation then analyzed. The resultant solution was filtered through Whatman filter paper, and the absorbance was measured at 228 nm using a UV-visible spectrophotometer [18]. To calculate the concentration of mirtazapine in the samples, the straight line equation obtained from regression anlaysis was used. The equation was obtained from the standard calibration plots, which were conducted in simulated nasal fluid, was well as a pH 6.4 phosphate buffer. The calibration curve of mirtazapine in the pH 6.4 phosphate buffer was found to obey Beer-Lambert’s law within the linearity range of 10–60 µg/mL, with a regression coefficient value of 0.992 at 228 nm, while in simulated nasal fluid, it was within the linearity range of 2–20 µg/mL, with a regression coefficient value of 0.991 at 228 nm.

#### 7.5.2. Drug Entrapment Efficiency

The drug entrapment efficiency of the mirtazapine nanoemulsion formulation was measured using the UV visible spectroscopic method. Each 1 mL sample was cooling centrifuged at 3500 rpm for 30 min. After centrifuge, the supernatant transparent layer was taken and diluted with 10 mL distilled water. The samples were measured at 228 nm using the UV-VIS spectroscopic method. Results were taken in triplicate, and the average was taken into consideration.

#### 7.5.3. In Vitro Drug Diffusion Studies

A Franz diffusion cell and a dialysis membrane were used in an in vitro drug diffusion study (Hi Media, molecular weight 5000 Daltons). Simulated nasal fluid (SNF) was used to saturate the dialysis membrane overnight. The dialysis membrane was then attached to the Franz diffusion cell’s upper (donor) and lower (receptor) compartments. On the donor compartment side of the dialysis membrane, the formulation corresponding to 7.5 mg of the dose was administered. An amount of simulated nasal fluid (SNF) equal to 18 mL was placed in the receptor compartment of the Franz diffusion cell. The diffusion cells (Remi, India) were maintained at 37 ± 0.5 °C throughout the experiment, with stirring happening at 600 rpm. At fixed time intervals, 1 mL of aliquots was withdrawn every 15 min, 30 min, 45 min, 60 min, 60 min, 120 min, 180 min, 240 min, 300 min, 360 min, and 420 min from the receptor compartment through a side tube, and an equal volume of SNF was replaced to maintain the sink condition [17,18,19] The samples withdrawn were analyzed through UV-visible spectrophotometry at 280 nm.

By dividing the slope of the steady state section of the line in the plot of drug amount penetrated per unit area of dialysis membrane verses time, the drug flux (g/hr/cm^2^) at the steady state was estimated.

#### 7.5.4. Ex Vivo Drug Diffusion Studies

Sheep nasal mucosa that had just been excised was used in a Franz diffusion cell for the ex vivo drug diffusion investigations of the mirtazapine nanoemulsion.

Sheep’s nasal mucosa was the subject of experiments on drug diffusion that were done ex vivo. The sheep nasal mucosa was taken from a freshly removed sheep’s nose at the butcher shop. Simulated nasal fluid (SNF) was used to soak this sheep’s nasal mucosa before it was glued to a Franz diffusion cell with a 3.14 cm^2^ diffusion area and then clamped. In the donor compartment, a formulation corresponding to a 7.5 mg dose was applied to the nasal mucosa, and simulated nasal fluid was placed in the receptor compartment. The diffusion cell was kept at body temperature throughout the experiment, and the stirring speed was kept at 600 rpm (Remi, India). At predetermined intervals, such as 15 min, 30 min, 45 min, 60 min, 120 min, 180 min, 240 min, 300 min, 360 min, and 420 min, the 1 mL aliquots were removed from the receptor compartment.

To keep the sink condition, the amounts of samples that were extracted were promptly replaced with equivalent amounts of SNF. At 228 nm, these samples were examined using a UV-visible spectrophotometer [19,20,21].

## 8. Fourier Transform Infrared Spectroscopy (FTIR) Studies

To evaluate any potential interactions that might have arisen during formulation between mirtazapine and other excipients, FTIR analysis was carried out. The infrared spectra of mirtazapine and thermotriggered nanoemulsion were acquired using a Bruker Alpha E, FTIR spectrometer (Bruker Alpha E, Opus-7.0.122), equipped with an ATR. They are typically evaluated using the single-reflection ATR accessories (attenuated total reflectance). The spectra were scanned in transmission mode spanning the 4000–650 cm^−1^ wavenumber range at room temperature.

### 8.1. Differential Scanning Calorimeter

To determine the physical condition of the medication in the nanoemulsion formulation, DSC experiments were carried out. A differential scanning calorimeter was used for the DSC measurements (Shimadzu, Kyoto, Japan, Thermal Analyzer DSC 60). Heat of fusion (enthalpy) (Hf) and peak transition temperature (Tm) were calculated and used in the analysis. For routine calibration, indium (Tm = 159.2 °C; Hf = 28.8 J/g) was utilized as the standard. Nitrogen (purity > 99.99%) was employed as the purge gas, and an empty aluminium pan served as the reference. Samples of 2.4–2.8 mg were weighed, put in open aluminium pans, and scanned over the temperature range of 30–350 °C at a rate of 10 °C/min.

### 8.2. Transmission Electron Microscopy

Transmission electron microscopy (TEM) (H-7500, Hitachi, Kyoto, Japan) was used to examine the morphology of the improved formulation in order to research the morphology of the resulting nanoemulsion. The nanoemulsion was placed on copper grids for observation after being dyed with 1% (*w*/*v*) phosphotungstic acid, and by examining the TEM images, the globule’s shape was discovered [22].

### 8.3. Stability Studies

#### 8.3.1. Thermodynamic Stability Tests

Selected formulations were subjected to different thermodynamic stability tests. Only those formulations that survived dispersion stability tests were selected for further study [21].

#### 8.3.2. Heating Cooling Cycle

All samples were placed between refrigerator temperatures of 4 °C and 45 °C for six cycles with storage at each temperature for not less than 48 h and were then studied. Those formulations that were stable at these temperatures were subjected to centrifugation.

#### 8.3.3. Centrifugation

Those formulations that passed were centrifuged at 3500 rpm for 30 min using centrifuge. The formulations that did not show any phase separation were taken for further tests.

#### 8.3.4. Freeze-Thaw cycle

Those samples that passed the centrifugation test were placed between –21 °C and +25 °C for three freeze-thaw cycles with storage at each temperature for not less than 48 h.

#### 8.3.5. Statistical Analysis

The statistical analysis was performed using one-way analysis of variance (ANOVA) and was used to compare the results of different formulations. A *p* value of 0.05 was considered to be statistically significant (GraphPad Prism version 6.03, Boston, MA, USA). The characterization data were expressed as the means of three experiments ± SD.

## Figures and Tables

**Table 1:** Physicochemical properties of nanoemulsion formulations.

Sr.No	Formulation	Physical Appearance	pH Range	Drug Content (%)	Drug Entrapment Efficiency	Viscosity (cps)	Stability
1	RD1	Transparent	6.2 ± 0.2	99 ± 0.5	82.56 ± 2	96 ± 2	Stable
2	RD2	Transparent	6.0 ± 0.2	98 ± 0.4	72.56 ± 2	91 ± 2	Stable
3	RD3	Transparent	6.1 ± 0.1	99 ± 0.2	72.56 ± 2	87 ± 2	Stable

**Table 2:** Particle size and zeta potential for developed formulations.

Formulation	Particle Size (nm) (Mean ± SD)	Zeta Potential	Poly Dispersity Index
RD1	42.60 ± 1.24	−6.58	0.345
RD2	135.20 ± 3.29	−2.25	0.435
RD3	52.44 ± 1.41	−0.193	0.299

**Table 3:** Composition of nanoemulsion formulations.

Sr.No:	Formulation	Oil Phase % *w*/*w*	S_mix_ % *w*/*w*	Water % *w*/*w*	Aqueous Phase % *w*/*w*		
					Poloxamer 407	Carbopol 934 P	PEG 6000
1	Thermotriggered nano emulsion (RD1)	3.846%	34.615%	61.53%	18.5%	0.1%	0.3%
2	Muco-adhesive nano emulsion (RD2)	3.846%	34.615%	61.53%	-	0.3%	-
3	Water based nano emulsion (RD3)	3.846%	34.615%	61.53%	-	-	-

## Data Availability

Not applicable.

## References

[1] Pires V.A., Fortuna A., Alves G., Falcão A. (2009). Intranasal Drug Delivery: How, Why and What for?. J. Pharm. Pharm. Sci..

[2] Xu J., Tao J., Wang J. (2020). Design and Application in Delivery System of Intranasal Antidepressants. Front. Bioeng. Biotechnol..

[3] Nguyen T.T., Maeng H.J. (2022). Pharmacokinetics and Pharmacodynamics of Intranasal Solid Lipid Nanoparticles and Nanostructured Lipid Carriers for Nose-to-Brain Delivery. Pharmaceutics.

[4] Devarajan V., Ravichandran V. (2011). Nanoemulsions: As modified drug delivery tool. Int. J. Compr. Pharm..

[5] Corazza E., di Cagno M.P., Brandl A.B., Abruzzo A., Bigucci T.C.F., Lupppi B. (2022). Drug delivery to the brain: In situ gelling formulation enhances carbamazepine diffusion through nasal mucosa models with mucin. Eur. J. Pharm. Sci..

[6] Cirri M., Maestrelli F., Nerli G., Mennini N., D’Ambrosio M., Luceri C., Mura P.A. (2021). Development of a Cyclodextrin-Based Mucoadhesive-Thermosensitive In Situ Gel for Clonazepam Intranasal Delivery. Pharmaceutics.

[7] Hari Kumar S.L., Singh V. (2012). Nanoemulsification—A novel targeted drug delivery tool. J. Drug Del. Therap..

[8] Mahajan S.H., Dinger B.S. (2011). Design and in vitro evaluation of nanoemulsion for nasal delivery of artemether. Indian J. Nov. Drug Del..

[9] Patel P.R., Joshi J.R. (2012). An overview on nanoemulsion: A novel approach. Int. J. Pharm. Sci. Res..

[10] Choudhary R., Goswami L., Kothiyal P. (2013). Preparation of nanoparticles loaded nasal gel of Mirtazapine for treatment of depression. J. Adv. Pharm. Sci..

[11] Benajeer S., Ramana K.V., Reddy V., Kumar A. (2012). New Simple UV Spectrophotometric Method for Determination of Mirtazapine in Bulk and pharmaceutical dosage forms. Int. J. Pharm. Sci. Res..

[12] Bahadur S., Pardhi D.M., Rautio J., Rosenholm J.M., Pathak K. (2020). Intranasal Nanoemulsions for Direct Nose-to-Brain Delivery of Actives for CNS Disorders. Pharmaceutics.

[13] Pathan S.A., Iqbal Z., Zaidi S., Talegaonkar S., Vohra D., Jain G.K., Azeem A., Jain N., Lalani J.R., Khar R.K. (2009). CNS Drug Delivery Systems: Novel Approaches. Recent Pat. Drug Deliv. Formul..

[14] Majithiya J.R., Ghosh P.K., Umrethia M.L., Murthy R.S.R. (2006). Thermoreversible-mucoadhesive Gel for Nasal Delivery of Sumatriptan. AAPS PharmSciTech.

[15] Thakkar H., Patel A., Chauhan N. (2014). Formulation and optimization of mucoadhesive microemulsion containing mirtazapine for intranasal delivery. Chron. Young Sci..

[16] Bernardi D.S., Pereira T.A., Maciel N.R., Bortoloto J., Viera G.S., Oliveira G.C., Filho P.A.D.R. (2011). Formation and stability of oil-in-water nanoemulsions containing rice bran oil: In vitro and in vivo assessments. J. Nanobiotechnol..

[17] Datta R., Bandyopadhyay A.K. (2005). Development of a new drug delivery system of diazepam with natural mucoadhesive agent from *Trigonella foenum-graecum* L.. J. Sci. Res..

[18] Kumar M., Misra A., Babbar A.K., Mishra A., Mishra P., Kamla P. (2008). Intranasal nanoemulsion based brain targeting drug delivery system of risperidone. Int. J. Pharm..

[19] Shah R.R., Chandrakant S.M., Patil S.S., Nilofar S.N. (2010). Preparation and Evaluation of Aceclofenac Topical Microemulsion. Iranian J. Pharm. Res..

[20] Subedia R.K., Ryoob J.P., Moonb C., Choi H.K. (2011). Influence of formulation variables in transdermal drug delivery system containing zolmitriptan. Int. J. Pharm..

[21] Kamble M.S., Sandeep M.D., Bhalerao K.K., Pravin D.C., Ashok V.B. (2012). Evaluation of brain targeting of drugs after administered intranasally. J. Biomed. Pharm. Res..

[22] Menaka M., Pandey V.P., Smith A. (2014). Colloidal dispersions as a potential nasal drug delivery system for ondansetron hydrochloride—In vitro and in vivo properties. Asian J. Pharm. Clin. Res..

